# Three-Dimensional Printing of Hydrogel Blend Tissue Engineering Scaffolds with In Situ Delivery of Anticancer Drug for Treating Melanoma Resection-Induced Tissue Defects

**DOI:** 10.3390/jfb15120381

**Published:** 2024-12-18

**Authors:** Xiao-Die Chen, Xin-Yang Zhang, Han-Qi Zhu, Helen H. Lu, Min Wang

**Affiliations:** 1 Department of Mechanical Engineering, The University of Hong Kong, Pokfulam Road, Hong Kong, China; xdchen@connect.hku.hk (X.-D.C.); u3008377@connect.hku.hk (X.-Y.Z.); zhuhanqi@hku.hk (H.-Q.Z.); 2 Department of Biomedical Engineering, Columbia University, 1210 Amsterdam Avenue, New York, NY 10027, USA; hl2052@columbia.edu

**Keywords:** three-dimensional printing, bioprinting, hydrogel blend, scaffold, drug delivery, tissue regeneration

## Abstract

Surgery is considered the gold standard for treating melanoma, but the high recurrence rate after surgery still remains as a major challenge. Therefore, using doxorubicin (DOX) as a model drug, this study investigated the 3D printing of anticancer drug-loaded hydrogel blend scaffolds for inhibiting post-operation melanoma recurrence and for promoting tissue regeneration. Three-dimensional printing could successfully produce methacrylate-modified chitosan (CSMA) and methylcellulose (MC) hydrogel blend scaffolds. Polymer blend inks exhibited satisfactory printability, and the printed porous scaffolds showed good biocompatibility and mechanical properties. Three-dimensionally printed DOX-loaded hydrogel scaffolds displayed controlled drug release, which may effectively prevent/impede tumor recurrence after surgery. Furthermore, combining 3D printing and bioprinting, DOX-loaded and rat bone marrow mesenchymal stem cell (rBMSC)-laden scaffolds were created for assessing local DOX delivery on healthy tissues. Within the 14-day culture period, rBMSCs encapsulated in multilayered scaffolds that were incorporated with DOX displayed rejuvenated cell viability. The 3D printed and bioprinted dual purpose hydrogel scaffolds have the promise of combating tumor recurrence and providing structural support for tissue regeneration.

## 1. Introduction

Melanoma, a serious form of skin cancer, has surged in recent decades [1]. The primary treatment for this dermatological disease is surgery, but it is not without drawbacks. The surgical treatment carries a substantial risk of relapse and also often results in significant skin defects [2,3]. Therefore, in clinical practice, surgical resection together with chemotherapy or radiotherapy is most commonly adapted. But normal chemotherapy or radiotherapy may face drug resistance or secondary adverse effects [4,5]. For example, doxorubicin (DOX), a commonly used anticancer drug, can cause nausea, vomiting, and cardiac and/or skin complications [6,7,8]. For chemotherapeutic agents that have high toxicity, attaining the desired high therapeutic concentration of the drug at the tumor site by increasing the dosage is still difficult to achieve [9,10]. Additionally, surgical resection can produce tissue defects that may require further reconstructive procedures. Consequently, a local, targeted drug delivery system (DDS) that can prevent tumor recurrence and also support tissue regeneration is highly desirable for treating melanoma after surgery.

Different materials (including hydrogels) and different drug delivery vehicles (including 3D-printed structures) have been utilized to produce localized therapies in integrative tumor treatment. These materials may modify the microenvironment at the surgical site. Furthermore, advances in 3D printing technologies have enabled the creation of multilayered drug delivery units embedded in regular porous structures that facilitate controlled and sustained drug release from these structures. In particular, extrusion 3D printing provides an efficient and versatile manufacturing platform for loading drugs into diverse polymers, ensuring the high loading efficiency of drugs and their controlled and sustained release [11,12]. Compared to traditional fabrication techniques, such as solvent casting-porogen leaching, gas foaming, emulsion freezing/freeze drying, phase separation, and electrospinning, the 3D printing of porous structures can achieve the desired properties, such as designed strut diameter, strut orientation, pore pattern, pore size, layered structure, etc., to create a conducive microenvironment for tissue regeneration [13,14]. Therefore, 3D printing can be an excellent route for obtaining usable devices for melanoma therapies in addition to providing desired tissue engineering scaffolds.

Hydrogels are water-containing gels composed of a network or networks of polymer chains that can be physically or chemically crosslinked [15]. They possess tunable physicochemical and mechanical properties, as well as permeability to nutrients and to waste products from encapsulated cells, mimicking the microenvironment of the extracellular matrix (ECM) of human body tissues [16,17]. The utilization of natural polymers such as pure collagen and chitosan (CS) has the advantages of promoting tissue regeneration and facilitating the fusion of interfaces through their ability to regulate cellular processes such as cell adhesion, proliferation, and differentiation. However, many biomedical hydrogels such as pure gelatin and alginate do not show adequate printability for the 3D printing of designed medical devices [18,19]. The insufficient mechanical properties of hydrogels can be addressed by printing a mixture of materials that combine one material to provide mechanical support and another material to enable cell encapsulation [20,21]. Common approaches in this route include co-printing cell-encapsulated inks with synthetic polymers such as poly(ε-caprolactones) (PCL). While synthetic polymers can be effective in providing the mechanical components to improve the mechanical properties of 3D-printed structures, these materials either require high temperatures for printing or are only soluble in organic solvents to prepare inks for 3D printing and, hence, they cannot be used directly for printing cell-laden structures [22,23].

CS is a low-cost, non-toxic, bioactive, bioadhesive, biodegradable, and cell-compatible cationic polysaccharide [24,25]. However, the mechanical strength of tissue engineering scaffolds made from CS is very low, which limits their clinical application. Moreover, CS is soluble in acidic environments, which poses further challenges for its in vivo use. Methacrylate-modified chitosan (CSMA) is a polymer in which methacrylate is functionalized to improve its solubility in water and to provide the UV crosslinking ability [26,27]. However, CSMA lacks printability at CSMA concentrations higher than 1.5%; and at CSMA concentrations lower than 1.5%, CSMA inks fail to form stable filaments during extrusion 3D printing due to its low viscosity [26]. Therefore, both the viscosity of CSMA inks and mechanical strength of 3D-printed CSMA-based scaffolds should be improved for CSMA use in 3D biomedical printing. Methylcellulose (MC) is a non-toxic and biodegradable polymer that has been used in a wide range of applications, including the use as thickeners [28,29]. Mixing CSMA and MC to form new inks should lead to improved printability for CSMA.

In the current study, a simple method for developing printable CSMA-based inks was investigated by the direct mixing of CSMA and MC hydrogels. The hydrogel blend inks were expected to provide adjustable viscosity and high printability as well as the structural stability of printed porous structures. The rheological properties of hydrogel blend inks, printing resolution, and properties such as surface morphology, water absorption, compression modulus, biodegradability, and thermal stability of 3D-printed hydrogel blend scaffolds were systematically studied. Furthermore, DOX was incorporated in hydrogel blend inks for constructing a localized anticancer DDS for post-operation melanoma treatments. The effect of DOX released from the DDS on the cell viability of rat bone marrow mesenchymal stromal cells (rBMSCs) was examined, assessing the potential of the DDS for providing integrative melanoma therapy.

## 2. Materials and Methods

### 2.1. Materials

CS, methacrylic anhydride (MA), acetic acid, gelatin (type A from porcine skin), 2-hydroxy-2-methylpropiophenone (a photoinitiator, known as I2959), phosphate-buffered saline (PBS) tablets, and bovine serum albumin (BSA) were Sigma-Aldrich (St. Louis, MO, USA) products. Dialysis tubing with a cutoff molecular weight of 12 kDa was also purchased from Sigma-Aldrich. Trypsinization (0.25% trypsin-EDTA, Gibco BRL, Grand Island, NY, USA) and live/dead viability/cytotoxicity kits were supplied by Thermo Fisher Scientific, Waltham, MA, USA. The synthesis of methacrylated gelatin (GelMA) is shown in the Supplementary Materials. All reagents were used as received without further purification.

### 2.2. Synthesis and Characterization of CSMA

**Figure 1:**
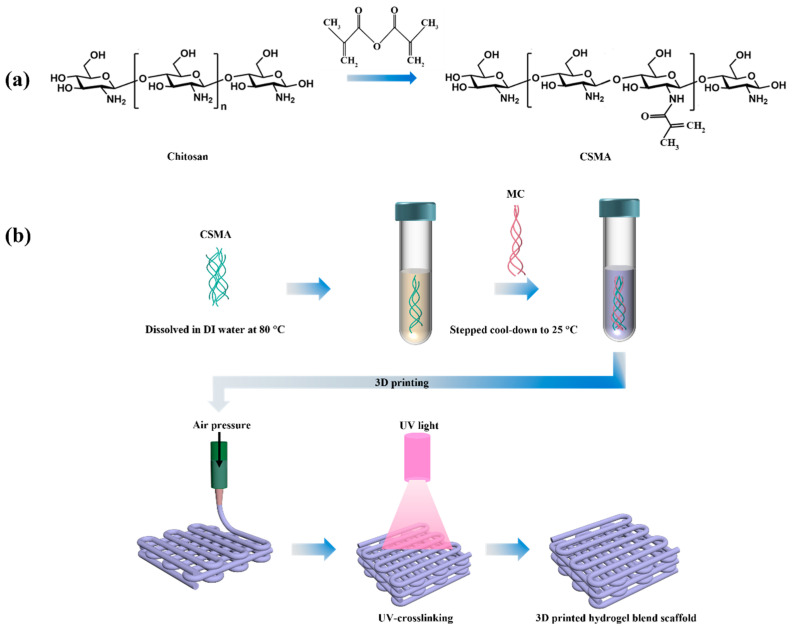
Illustrations for (**a**) the chemical reaction to obtain CSMA and (**b**) 3D printing of hydrogel blend scaffolds.

CSMA was synthesized by single-step chemoselective N-acylation between CS and MA according to Li et al. [27] with minor modifications. In aqueous environments, the amino group demonstrates significantly greater reactivity compared to the primary hydroxyl group in acylation modifications [15,30]. It was believed that acylation predominantly took place on the amino group in this work. As shown in Figure 1a, MA was added slowly drop-wise to a 1% (*w*/*v*) CS acetic acid solution at 60 °C with a ratio of 4:1 for acid anhydride to amino group and stirred for 3 to 4 h. Afterwards, the mixture was dialyzed (with MW 12 kDa cut-off) for a week at 60 °C. Finally, the solution was freeze-dried and kept at −20 °C until use.

^1^H NMR spectra were recorded on a Bruker Avance III 400 spectrometer from Bruker Ltd., Billerica, MA, USA (400 MHz) at 25 °C, using D_2_O δ(^1^H) (4.79 ppm) as the solvent. The degree of substitution (DS) of CSMA was calculated by Equation (1).

(1) $$DS=\frac{A_{H(5.5\&5.7)}/2}{A_{H(2.5-4.1)}/5}\times 95\%$$

where A_H(5.5&5.7)_ and A_H(2.5–4.1)_ are the areas of methylene proton peak (Hg) at 5.46 and 5.68 ppm (the ring proton (Hc~Hf) peak of GlcN residues at 2.52–4.18 ppm), respectively [27,31].

### 2.3. Preparation of Hydrogel Blends as Printing Inks

An amount of CSMA was dissolved in deionized (DI) water to obtain a 1% (*w*/*v*) CSMA solution at 80 °C. MC powders were then added to make CSMA/MC hydrogel blends having MC concentrations of 6% (*w*/*v*), 8% (*w*/*v*), 10% (*w*/*v*), and 12% (*w*/*v*). The mixtures were subjected to continuous magnetic stirring until the MC powder was uniformly dispersed. The mixtures were cooled down to 40 °C and the photoinitiator [0.01% (*w*/*v*)] was added. They were gradually cooled down to room temperature, at which point the MC started to hydrate, resulting in an increase in the viscosity of the mixtures. Finally, the mixtures were stirred at room temperature overnight to obtain completely dissolved CSMA/MC hydrogel blends with different MC contents. The prepared CSMA/MC hydrogel blends with different MC amounts were designated as CSMA-6MC, CSMA-8MC, CSMA-10MC, and CSMA-12MC, as shown in Table 1.

### 2.4. Printability

The rheological properties of the CSMA/MC hydrogel inks were investigated by using a rotational rheometer (MCR302, Anton Paar, Graz, Austria) with 25 mm diameter parallel plates and 0.55 mm measurement gap. Three types of tests were conducted at 25 °C: (1) shear thinning tests, (2) angular frequency sweep tests, and (3) thixotropy tests. For studying the shear thinning behavior of inks, the viscosity of hydrogel inks was estimated at shear rates from 0.001 to 1000 s^−1^. For angular frequency sweep tests, strain sweeps from 0.1% to 100% at a frequency of 1 s^−1^ were performed to locate the linear viscoelastic region (LVR). From LVR, a strain of 1% was selected for angular frequency sweep at an angular frequency range of 0.1–100 rad^−1^. Thixotropy tests were performed in three steps to assess the recoverability of CSMA/MC hydrogels. In step I, a shear rate of 0.1 s^−1^ was applied to hydrogel samples for 60 s to observe the state of the hydrogel without shear force, simulating the situation prior to 3D printing. In step II, the shear rate was instantaneously and substantially increased and held for 10 s to observe the shear state of the hydrogel subjected to shear, simulating the situation during 3D printing. In step III, the shear rate was restored to 0.1 s^−1^ and held for 60 s to observe the recovery of viscosity of the hydrogel after shear was removed, simulating the situation after printing.

To investigate the shape fidelity of CSMA/MC hydrogel scaffolds, hydrogel inks were printed into monolayer square grids (20 × 20 mm^2^, line spacing of 1.4 mm) on glass slides using a 3D bioprinter (3D Discovery Evolution, regenHU Ltd., Fribourg, Switzerland). After printing was completed, the shape of grids was photographed using an optical microscope (Leica Microsystems, Wetzlar, Germany). The strut diameter of each printed grid was analyzed at 5 different locations using the ImageJ software bundled with 64-bit Java 8 (https://imagej.net/ij/, accessed on 10 October 2024). The printability (*Pr*) of a hydrogel was determined by using Equation (2):

(2) $$Pr=\frac{L^{2}}{16A}$$

where L and A are the perimeter and area, respectively, of a single printed grid and were measured using the ImageJ software bundled with 64-bit Java 8 [32].

### 2.5. Three-Dimensional Printing of Hydrogel Blend Scaffolds

All hydrogel scaffolds were printed using the regenHU 3D bioprinter (3D Discovery Evolution, regenHU Ltd., Fribourg, Switzerland). The extrusion rate was selected by changing the air pressure until the hydrogel ink could be extruded uniformly as long filaments, which became struts in printed scaffolds. The layer thickness was set at 0.15 mm, the printing speed was 6 mm/s, and the nozzle inner diameter was 21 G. Subsequent to 3D printing, the printed scaffolds were exposed to a UV light (wavelength of 365 mm) from 5 to 30 min depending on the experiments. Figure 1b illustrates the 3D printing process of hydrogel blend scaffolds.

### 2.6. Characterization of Hydrogel Blend Scaffolds

The strut morphology and structure of printed hydrogel scaffolds was characterized using SEM (Hitachi S3400 SEM, Tokyo, Japan). Hydrogel scaffold samples were frozen at −70 °C for 12 h and then freeze-dried for 1 day. They were sputter-coated with a thin layer of gold before SEM observation. The SEM acceleration voltage was 20 kV.

Chemical structures of hydrogels in scaffolds were analyzed using Fourier-transform infrared spectroscopy (FTIR) (PerkinElmer, Waltham, MA, USA). Scaffold samples (1 × 1 cm^2^) were placed on a sample holder and analyzed in the wave number range from 400 to 4000 cm^−1^. FTIR data were obtained by accumulating more than 64 scans.

Structural changes in the hydrogels in the printed scaffolds were investigated using Rigaku from Tokyo, Japan (SmartLab: Automated Multipurpose X-ray Diffractometer) equipped with Ge (220)×22-bounce monochromators for filtering Kα2. Analyses were conducted through the monochromic XRD of the wavelength 0.154 nm with a 2θ glancing angle from 2° to 60° and a step size of 0.0003°.

The thermal properties of hydrogel scaffolds were studied using thermogravimetric analysis (TGA) (DZ TGA101, Nanjing Dazhan, Nanjing, China) from ambient temperature to 800 °C at the 20 °C/min heating or cooling rate under a nitrogen atmosphere.

For determining mechanical properties, cylindrical hydrogel samples with a diameter of 4.5 mm and a height of 10 mm were made. They were tested under compression using a tabletop mechanical testing machine (Model 5848, Instron Ltd., Boston, MA, USA) at the testing speed of 4 mm/min. A digital micrometer (Mitutoyo, Kanagawa, Japan) was used to measure the diameter and height of each sample. The compression limit was set at 98% strain to avoid crashing the load cell. The compressive modulus was calculated from the slope of the stress–strain curve for each sample.

The water absorption ability of hydrogels with different MC concentrations was measured using a gravimetric method. Briefly, cylindrical samples of crosslinked hydrogels were freeze-dried. They were rehydrated with PBS, weighted using an analytical balance, and then immersed in PBS at 37 °C for 48 h. Subsequently, the samples were removed from the PBS, surface-dried with tissue paper, and weighted. The water uptake of each sample was calculated using Equation (3):

(3) $$Water\;uptake\;(\%)=\frac{W_{s}-W_{d}}{W_{d}}\%$$

where W_d_ and W_s_ are the weights of the dry and swollen samples, respectively.

The swelling of hydrogels with different MC concentrations was studied by measuring the volumetric changes in samples. Cylindrical samples of crosslinked hydrogels were freeze-dried and then rehydrated in PBS at 37 °C for 48 h. The swelling ratio (SR) was determined via Equation (4):

(4) $$SR=\frac{V_{s}-V_{d}}{V_{0}}\%$$

where V_d_ and V_s_ are the volumes of the dry and swollen samples, respectively.

For investigating the biodegradation of hydrogels, each cylindrical sample was immersed in 5 mL PBS (pH 7.4) supplemented with 0.02% sodium azide at room temperature until it reached a constant weight and the weight was recorded. Then, it was immersed in 5 mL PBS (pH 7.4) supplemented with 0.02% sodium azide in a sealed test tube that was placed in a water bath at 37 °C. The PBS in the test tube was refreshed every 3 days. At each predetermined time point (1, 2, 3, and 4 weeks), the sample was taken out, washed with DI water, and weighted. Finally, the weight losses (WLs) over time were calculated via Equation (5):

(5) $$WL=\frac{W_{c}-W_{de}}{W_{c}}\%$$

where W_c_ and W_de_ are the weight of a constant weight and degradation weight at predetermined time point of the samples, respectively.

### 2.7. Fabrication of DOX-Loaded CSMA/MC Hydrogels and In Vitro Drug Release Study

To prepare DOX-loaded CSMA/MC hydrogels, 100, 200, and 500 μg/mL of DOX were added to CSMA-8MC hydrogel inks. After casting the CSMA-8MC hydrogel into a mold with a cylindrical chamber of a diameter of 9.7 mm and a height of 10 mm, UV crosslinking was performed for 10 min. To study the in vitro release behavior of DOX, cylindrical hydrogel samples loaded with DOX were immersed in test tubes containing 12 mL of PBS per tube. The sealed test tubes were then placed in a shaking water bath at 37 °C. At predetermined time points, 2 mL of the immersing liquid was collected from each tube, with the tube being re-filled with 2 mL of fresh PBS. An enzyme-linked immunosorbent assay plate reader (UVM 340, Asys HiTech GmbH, Salzburg, Austria) was used to obtain optical density (OD) values at 480 nm absorbance, which were associated with the amounts of DOX in the extracted immersing liquid. Each release amount of DOX was determined using the standard concentration curve established in the current drug release study. Finally, cumulative release curves of DOX were constructed for different types of hydrogel samples.

### 2.8. Cells and Cell Culture

Rat bone marrow-derived mesenchymal stem cells (rBMSCs) were used in the current study. They were cultured with Dulbecco’s modified Eagle’s medium (DMEM, Gibco BRL, Grand Island, NY, USA) supplemented with 10% fetal bovine serum (FBS, Gibco BRL, Grand Island, NY, USA), and 100 U ml^−1^ penicillin–streptomycin (Gibco BRL, Grand Island, NY, USA) in a humidified incubator at 37 °C with 5% CO_2_. The culture medium was refreshed every 2 days.

### 2.9. Preparation of Bioinks Containing rBMSCs

Bioinks based on GelMA and gelatin were prepared following the procedures used by Lai et al. [33]. GelMA is a photopolymerizable gelatin-based hydrogel functionalized with methacrylic anhydride. GelMA preserves the functional amino acid sequence of gelatin, such as the gelatin arginine–glycine–aspartic acid (RGD) sequence, which can promote cell adhesion and proliferation [34]. In addition, GelMA hydrogels are thermally reversible, i.e., GelMA hydrogels are liquid at 37 °C and gel at below 20 °C, and are commonly employed for bioprinting work. After incorporating cells in the ink at 37 °C, bioprinting could be performed at 20 °C, followed by photo-crosslinking, which facilitates cell incorporation and scaffold formation in 3D bioprinting. To prepare bioinks, 0.1 g GelMA and 0.1 g gelatin were added into 2 mL PBS, and after being fully stirred, 10 µL of I2959 (the photoinitiator) was added to the ink for the subsequent UV crosslinking of the hydrogel. GelMA/gelatin blended hydrogels were sterilized by ^60^Co irradiation at room temperature for 20 min. The sterilized hydrogels became liquid when heated to 37 °C and 2 × 10 rBMSC cells were added to the liquid, which was thoroughly mixed by gentle mechanical stirring for 30 s. The rBMSC-containing bioink was loaded into a 5 mL syringe and kept at 4 °C for about 3 min to convert the liquid bioink to a gel state for subsequent bioprinting.

### 2.10. Bioprinting of rBMSC-Laden Multifunctional Scaffolds

Gelatin and GelMA with a substitution degree of 60.69% (Figure S1) were used to produce bioinks. For the 3D bioprinting of cell-laden multifunctional scaffolds, CSMA/MC@200DOX hydrogel and rBMSC-laden GelMA/gelatin hydrogel were printed at 20 °C alternately using two printing heads of the 3D bioprinter (3D Discovery Evolution, regenHU Ltd., Fribourg, Switzerland). In this hybrid 3D bioprinting process, CSMA/MC@200DOX ink was loaded into syringe 1 and GelMA/gelatin-rBMSCs bioink was loaded into syringe 2. The printing parameters for the CSMA/MC@200DOX ink were the same as those stated in Section 2.5. The printing parameters for the GelMA/gelatin-rBMSC bioink were 0.031 mm/s for piston speed, 12 mm/s for printing speed, syringe temperature of 20 °C, layer thickness of 0.18 mm, and 25 G for the nozzle inner diameter of the printing head. A layer of CSMA/MC@200DOX scaffolds was printed on a six-well culture plate first, followed by the continuous placement of GelMA/gelatin-rBMSC struts between the CSMA/MC@200DOX struts. After one scaffold layer printing was completed, UV crosslinking (365 nm) was performed at the power of 360 mW for 2 min. This 3D printing/bioprinting crosslinking process was repeated for producing subsequent scaffold layers until the whole scaffold was built. Finally, the Dulbecco’s modified Eagle’s medium, (DMEM, Gibco BRL, Grand Island, NY, USA) supplemented with 10% fetal bovine serum (FBS, Gibco, USA) and 100 U ml^−1^ penicillin–streptomycin (Gibco BRL, Grand Island, NY, USA), was added into the 6 wells, and the multifunctional scaffolds were cultured in a humidified incubator at 37 °C with 5% CO_2_.

### 2.11. Cell Viability

The live/dead assay was used to study the viability of rBMSCs in cell-laden scaffolds at predetermined time points, i.e., immediately after printing and 1d, 3d, 7d, and 14d of culture. Cell-laden scaffolds were washed with sterilized PBS and then incubated with PBS containing 4 µM ethD-1 and 2 µM calcein AM for 20 min at room temperature, being protected from light. Live and dead cells were stained green by calcein AM and red by EthD-1, respectively. A fluorescence microscope (Leica DMi8, Wetzlar, Germany) was used to obtain fluorescence images of scaffolds, with the incorporated cells being stained. Cell viability was quantified by the ratio of live cells to all cells observed in the scaffold.

### 2.12. Statistical Analysis

All experiments in this study were conducted for a minimum of three times, with the data being presented as mean ± standard deviation. Data analysis was conducted using a one-way analysis of variance (ANOVA), with statistical significance levels set at * *p* < 0.05, ** *p* < 0.01, and *** *p* < 0.001.

## 3. Results and Discussion

**Figure 2:**
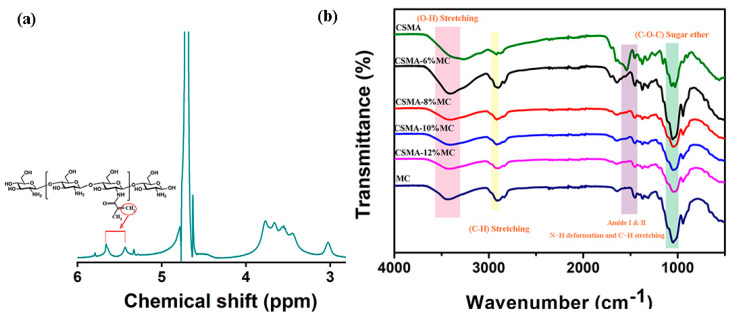
Spectroscopical analysis of polymers and scaffolds: (**a**) ^1^H NMR spectrum of synthesized CSMA; (**b**) FTIR spectra of hydrogel scaffolds.

CSMA was prepared by N-acylation reaction. The DS value for CSMA synthesized in the current study was 35.06% according to ^1^H NMR measurements (Figure 2a) and Equation (1), which was similar to Li et al.’s result [27]. The FTIR spectra of hydrogel scaffolds together with those for virgin CSMA and MC are shown in Figure 2b. Owing to the different scaffold compositions, the spectra for scaffolds showed highly similar band patterns with small differences. Amide-I and amide-II bands were observed at ~1641 cm^−1^ in all scaffolds due to N-H deformation and C-H stretching. Absorption bands at 2900 to 2800 cm^−1^ corresponded to C-H stretching vibrations, bands at 1100 to 1000 cm^−1^ originated from C-O-C stretching vibrations, the band corresponding to N-H stretching in CSMA was observed at 3260 cm^−1^, and the band for O-H stretching in MC appeared at 3430 cm^−1^ [35]. With the increase in the MC content in the CSMA/MC scaffolds, the N-H peak of CSMA in the FTIR spectra gradually diminished; and for the CSMA-12MC scaffolds, the characteristic N-H peak could not be observed, suggesting the high amount MC. The absorption band at 1061 cm^−1^ for CSMA was slightly shifted to 1053 cm^−1^, which was due to the interaction between CSMA and MC.

Through rheological analyses, shear thinning behavior, LVR and thixotropic property of printing inks were investigated. In shear-thinning experiments, it was found that the viscosity of ink samples decreased with an increase in the shear rate, revealing the shear thinning behavior of the inks, which would make the inks flow easily through the nozzle in the printing head during the 3D printing process and cause a stable formation of filament after extrusion from the nozzle [36]. In addition, with the increase in the MC content, the viscosity of inks increased gradually, indicating that MC served well as a thickener in the printing inks. The increase in the MC concentration increased the number of sites where CSMA and MC could interact, which contribute to improving the printing resolution of scaffolds. From angular frequency sweep tests, it was found that the storage modulus G′ of all inks were higher than their loss modulus G″, indicating that all inks were in a gel-like state [37]. In thixotropic property tests, all inks showed a stable viscosity under a low shear rate of 0.1 s^−1^ in step I. Their viscosity then decreased sharply to a low viscosity level under a high shear rate of 400 s^−1^ in step II. In step III when the high shear rate was removed, it finally recovered to a viscosity level comparable to their initial value, indicating a good thixotropic property for all inks tested. These results suggest that the polymer networks were disentangled during shearing and that they were rebuilt with the recovered viscosity after shearing [14]. This rheological behavior would increase the high fidelity of 3D-printed CSMA/MC hydrogel scaffolds.

**Figure 3:**
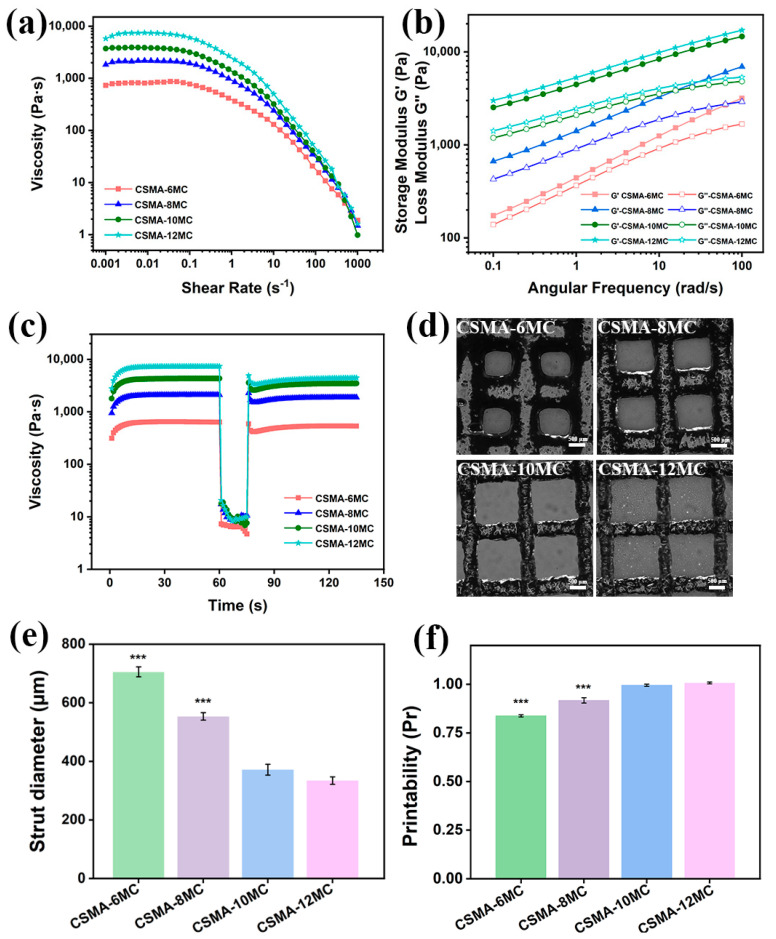
Rheological properties and printability of CSMA/MC inks at room temperature: (**a**) shear viscosity, (**b**) storage modulus (G′) and loss modulus (G″), (**c**) thixotropic property, (**d**) printed monolayer grids, (**e**) struct diameters of printed grids, and (**f**) *Pr* value for each hydrogel blend scaffold (* *p* < 0.05, ** *p* < 0.01, and *** *p* < 0.001). XRD diffraction patterns were obtained for investigating structural changes in printed hydrogel blend scaffolds. As shown in Figure S2, CSMA exhibited a broad hump peaking at 2θ of 19.97°, while MC showed a barely visible hump at 2θ of 8.15°, which reflected the amorphous nature of these materials [38]. The hydrogel blend scaffolds displayed both humps but shifted the hump from 8.15° to 9.40° (Figure 4a), suggesting the uniform distribution of MC in hydrogel blends and the interaction between CSMA and MC.

The printing of the monolayer grids of the hydrogel blends was conducted to investigate the printability of the hydrogel blend inks and the fidelity of the printed structures. Figure 3d shows optical microscope images of printed monolayer hydrogel blend scaffolds. CSMA-6MC hydrogel could hardly provide a grid-like structure after printing and the printed filaments showed a low resolution with an average diameter of about 0.7 mm. When the MC concentration was increased to 12%, the strut diameter decreased drastically to 0.33 mm and the printed structures were observed to be stable.

In 3D printing, for the ideal printability of an ink, the *Pr* value of a printed scaffold is 1. In the current study, the *Pr* values as calculated through Equation (2) were 0.84, 0.92, 1.00, and 1.00 for the CSMA-6MC, CSMA-8MC, CSMA-10MC, and CSMA-12MC hydrogel blend scaffolds, respectively. The increase in the MC concentration in the inks significantly improved the printability of the hydrogel blend inks. For CSMA-10MC and CSMA-12MC, the shapes of the printed grids were highly consistent with the designed pattern for printing, and each strut in the grids exhibited a high resolution. Increasing the MC concentration increased the viscosity and, hence, the printability of the hydrogel ink. Although the 12% MC concentration in the inks led to decrease in the strut diameter in the printed grids, *Pr* was already nearly 1 when the MC concentration reached 10%. In addition, the 12% MC concentration in the ink significantly increased the difficulty in preparing homogenous inks. Therefore, no significant benefits could be gained when the MC concentration was raised above 10%.

**Figure 4:**
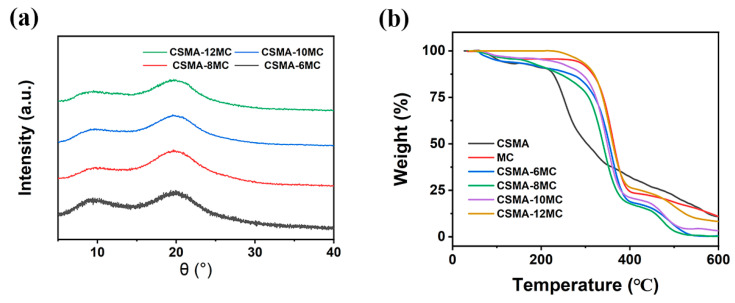
(**a**) XRD patterns and (**b**) TGA curves of the hydrogel blend scaffolds of different MC concentrations.

TGA can be used to study the thermal stability of polymers for 3D printing [39]. The thermal stability of CSMA, MC, CSMA-6MC, CSMA-8MC, CSMA-10MC, and CSMA-12MC scaffolds was investigated using TGA. The results in Figure 4b show that the addition of MC had a significant effect on the thermal degradation behavior of CSMA. With the increase in the MC content, the thermal degradation temperature of the CSMA-MC blend scaffolds gradually increased, which was particularly obvious for CSMA-12MC scaffolds, where the thermal degradation temperature increased to about 230 °C. Additionally, the addition of MC also increased the residual amounts of CSMA-MC blend scaffolds at 600 °C. This was because MC was relatively stable even at 600 °C. All CSMA-MC blend scaffolds underwent three stages of weight loss during TGA heat-up. At 50–230 °C, the weight loss was due to water evaporation, while the second weight loss at 230–400 °C was due to the thermal degradation of CSMA [40]. The third weight loss at 420–520 °C was due to the generation of by-products at high temperatures. These TGA results show that the addition of MC improved the thermal stability of the CSMA-MC scaffolds.

**Figure 5:**
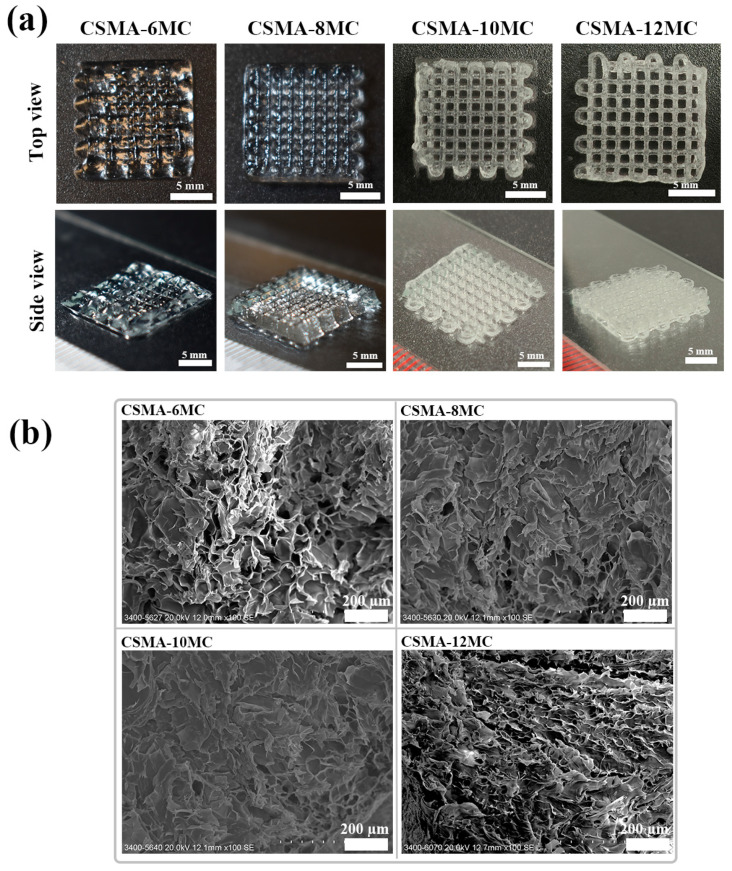
(**a**) Photos of 3D-printed 5-layer hydrogel blend grid structures. (**b**) SEM images showing the microporous surface morphology of struts of 3D-printed hydrogel blend scaffolds.

The CSMA/MC inks could be successfully printed into 3D five-layer hydrogel structures with a lattice-like pattern (Figure 5a). Except for the CSMA-6MC scaffolds, the other three types of scaffolds showed a tightly stacked structure. From the front view of the ten-layer scaffolds (Figure S3), it was observed that, except for CSMA-6MC, the other scaffolds show fully interconnected lattice structures with no internal pore collapses. The strut resolution of five-layer grid structures increased with the increase in the MC concentration. The excellent rheological property of CSMA/MC blend inks provided good ink adjustability, allowing the printing of multilayered lattice structures without collapsing, which ensured the structural integrity of printed structures as tissue engineering scaffolds. Increasing the MC concentration in inks facilitated the retention of the printed shape during and after 3D printing. This could be attributed to the increased number of available interaction sites between MC and CSMA, which resulted in high printability and adequate viscoelastic behavior to maintain well-defined shapes and robust structures after 3D printing.

SEM was used to examine the morphology and structure of the 3D-printed scaffolds. SEM images revealed the open porous structures (Figure 5b). It is apparent that all hydrogel blend scaffolds had a homogeneous porous structure. The microporous strut surface of hydrogel scaffolds could promote cell adhesion while macropores in scaffolds would enhance nutrient diffusion [41].

The limited printability of CSMA for 3D printing has been a challenge. Jia et al. attempted to create 3D-printed wound dressings using 4% CSMA (dissolved in DI water) but encountered issues with accuracy and stacking [42]. They then used PCL as a substrate to provide the support, but the printed CSMA structures still lacked improvement [43]. Increasing the CSMA concentration may improve its 3D printing and, hence, Zanon et al. tried using 5% CSMA in 2% acetic acid to print a CSMA hydrogel grid structure with four layers [44]. But they still encountered low printability with irregular holes, while the use of acetic acid was not conducive to the in vivo application of 3D-printed scaffolds. Optimizing the synthesis method has also been investigated with the aim to improve the printability of CSMA. Cebe et al. printed a high-resolution multi-layer scaffold by modifying CS with methyl methacrylate and laponite nanosilicates in situ [45]. In addition to these efforts, blending CSMA with other components has been used to improve printability. Blending CSMA with polyvinyl alcohol (PVA) improved printability but hindered extrusion. Therefore, Zhang et al. repeatedly squeezed the ink through a nozzle of a specific diameter to control the average particle size of the ink before printing [31]. Finally, the ink was successfully printed into a mouse-size thigh bone model and a human ear model. Physical mixing to increase the viscosity of CSMA inks appears to be a more feasible route. Osi et al. printed a scaffold with a height of 5 mm using the hydrogel ink with 1% CSMA, 20% GelMA, and 0.5% nanohydroxyapatite (nano-HAp) [26]. In our work, we successfully made a user-friendly ink through a simple process of physical blending. By dissolving the polymer in DI water and avoiding an acidic environment like acetic acid, we obtained an ink with excellent printability. This ink had excellent shear-thinning properties, making it easy for smooth extrusion through a small-diameter nozzle without the need for pre-printing screening of inks, which had to be adopted by other research groups in their studies in order to have smooth printing. The printed hydrogel scaffolds displayed the desired structural integrity. In short, the current study developed a general strategy to prepare CSMA-based inks with favorable printability, which could be successfully printed into CSMA-based multilayered hydrogel scaffolds.

**Figure 6:**
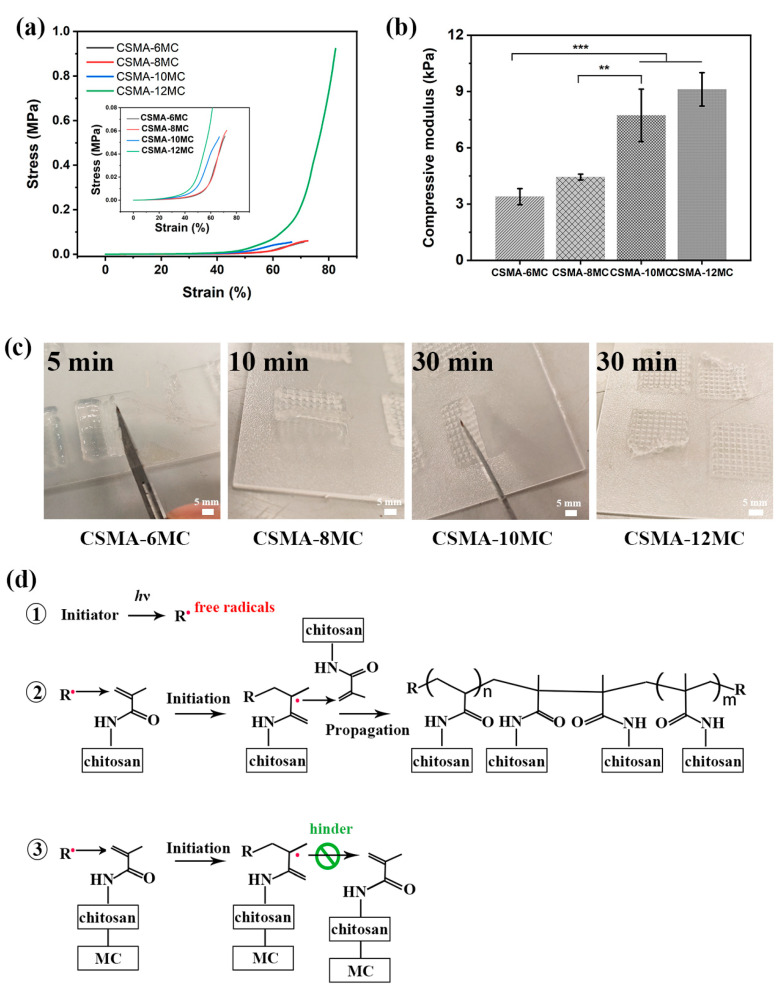
(**a**) Compressive stress–strain curves (insert: the magnified area of curves showing the starting points for their rises) and (**b**) compressive modulus of hydrogel samples with different MC concentrations (** *p* < 0.01, and *** *p* < 0.001); (**c**) UV crosslinking time of hydrogel blend scaffolds and (**d**) chemical reaction in UV crosslinking in the CSMA-MC hydrogel blends of 3D-printed scaffolds.

Tissue engineering scaffolds with suitable mechanical properties provide the mechanical stability for cell attachment and guide cells for their appropriate behavior (growth, proliferation, and differentiation). Through compression tests, compressive stress–strain curves of hydrogel samples were obtained and, subsequently, the compressive modulus of these samples was calculated (Figure 6a,b). Increasing the MC concentration from 6% to 12% increased the compressive modulus of hydrogel blends from about 3.40 kPa to 9.11 kPa. These modulus values were similar to the modulus of previously reported materials used for treating melanocytoma; and they also showed the effectiveness of MC addition in improving the mechanical properties of composite hydrogels [46,47]. Due to chain entanglement between CSMA and MC, the polymer networks in hydrogel blends could withstand higher stresses from external loads. However, as the concentration of MC increased to 10% and 12%, the yield stress of hydrogel blends appeared to decrease (Figure S4). For CSMA-6MC and CSMA-8MC, the compressive stress at failure was similar. To find explanations for this phenomenon, the crosslinking time for hydrogel scaffolds containing different MC amounts was investigated (Figure 6c). For photo-crosslinked hydrogels, increasing the photo-crosslinking time could improve the crosslinking efficiency and maintain the integrity of the structure [33]. As the MC concentration increased, hydrogel scaffolds required longer crosslinking time. It was noted that, even after 30 min of UV crosslinking, hydrogel scaffolds with 12% of MC still could not be easily taken up from the substrate. Figure 6d shows the chemical reaction in UV crosslinking for CSMA/MC scaffolds. After UV irradiation, the free radicals generated by the photoinitiator initiated the chain polymerization of the methacryloyl substituent [34]. In the current study, we hypothesized that increased entanglements between MC chains and CSMA chains may hinder reaction propagation. The number of elastic active connections per unit volume of the polymer network, n_e_ (in mol/m^3^), is calculated as follows [48,49]:

(6) $$n_{e}=\frac{G_{e}}{RT}$$

where R is the universal gas constant (8.314 J/(mol K)) and T is the measurement temperature (298 K). The equilibrium shear elastic modulus G_e_ corresponds to the plateau value of G′ measured by frequency (Figure 3b). The data presented in Figure S5 illustrated the n_e_ values of the four hydrogel formulations. The analysis of Figure S5 suggested that increased MC levels correspond to an increased presence of entrapped entanglements within the hydrogel network [49]. This observation served to substantiate our initial hypothesis. However, further investigations will be conducted to clarify this hypothesis.

**Figure 7:**
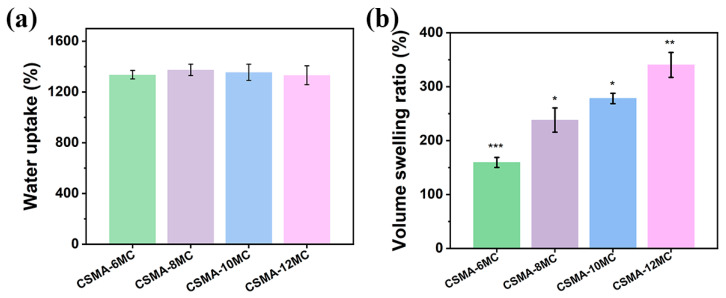
(**a**) Water uptake of and (**b**) volume change in the hydrogel blends with different MC concentrations (* *p* < 0.05, ** *p* < 0.01, and *** *p* < 0.001).

In general, the water content has a significant effect on the physical properties and also the structural fidelity of hydrogels [26,50]. As shown in Figure 5b, the increase in the MC concentration did not significantly affect the pore size of the hydrogel blend scaffolds. In this paper, it was observed that the water absorption capability of four hydrogel blends was also not significantly different (Figure 7a). The swelling of a hydrogel caused by water molecules can create voids in the gel structure, allowing the release of drugs or cells encapsulated in the hydrogel [51]. In swelling tests, after 48 h of immersion, an increase in the MC concentration in the hydrogel blends increased the volume swelling ratio value of the hydrogel samples (Figure 7b). The swelling ratio of hydrogels could be significantly affected by strong interactions between polymer chains in hydrogels or hydrogel blends [52]. As shown earlier, the addition of MC into CSMA increased the entanglement between these two types of polymer chains and, hence, increased the swelling ratio. The swelling of synthetic polymer gels is usually described by the classical Flory–Rehner theory, which states that swelling is a thermodynamic property determined by two independent factors: osmotic pressure and elastic pressure [53]. This is also applicable in our hydrogel system.

**Figure 8:**
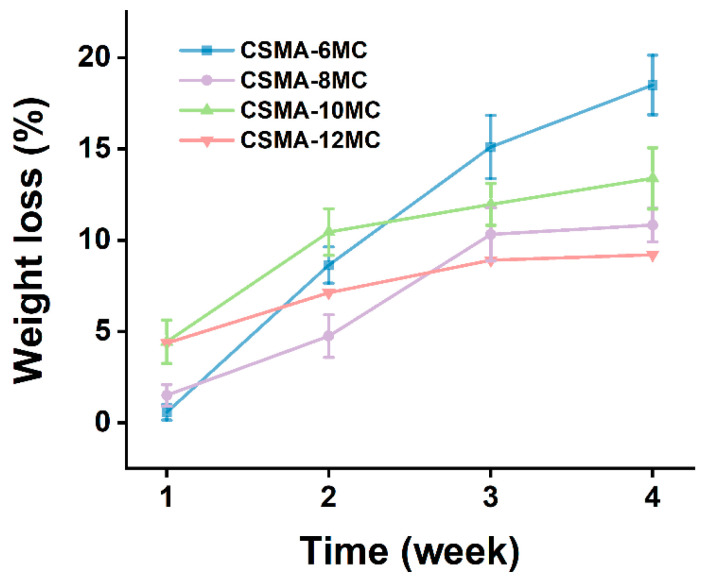
Weight loss vs. immersion time for the hydrogel blends with different MC concentrations in the in vitro biodegradation tests.

To investigate the biodegradation behavior of the CSMA/MC hydrogel scaffolds, the weight loss of the hydrogel samples during the in vitro degradation test was recorded, and the results are shown in Figure 8. The weight loss of the hydrogel samples showed a similar increasing trend over the immersion time. In the 4-week degradation test period, under the same PBS soaking condition, the weight loss of CSMA-8MC was lower than that of CSMA-6MC. The degradation of CSMA-6MC increased first; and it then slowly decreased in the fourth week. The weight loss after four weeks of immersion was 33.00 ± 5.34% for the CSMA-6MC samples. The degradation of CSMA-8MC was slightly different, with a slight increase in degradation after two weeks of immersion. Its weight loss was 23.70 ± 1.22% after four weeks of immersion.

**Figure 9:**
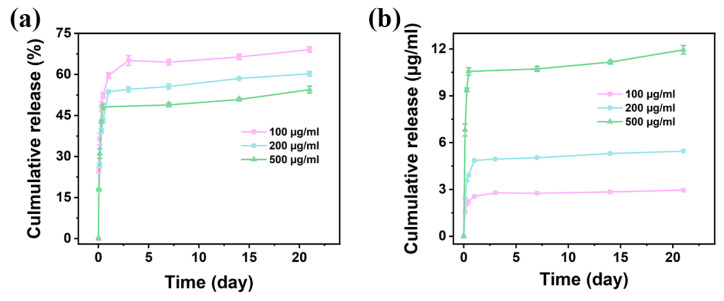
In vitro DOX release behavior of the CSMA-8MC hydrogel blend with different DOX concentrations: plots in terms of (**a**) cumulative percentage and (**b**) concentrations.

Ideally, a DDS should be capable of loading a sufficient amount of drug for its manufacture and then release the drug in vivo in a controlled and sustained manner [54,55]. Additionally, in light of the inherent resistance to drugs by melanoma, using DOX at a low dosage to combat residual melanoma cells following surgical resection may not work. Therefore, in the current study, moderate DOX concentrations of 100, 200, and 500 μg/mL were used to investigate their effect in combating this cancer. The drug release results are shown in Figure 9. The CSMA-8MC formulation was selected as the DDS due to its comparatively favorable printability and rapid crosslinking properties. The amount of released DOX increased with an increase in the DOX concentration from 100 to 500 μg/mL in the CSMA-8MC hydrogel. The DOX release curves of the three types of DOX-incorporated hydrogels revealed a two-stage release behavior. In the first stage (i.e., in the 1st day), a rapid release of DOX was observed in all three types of hydrogels with different concentrations of DOX. The rapid release of DOX may be attributed to the dissociation of DOX molecules located on the surface of hydrogel samples after immersion in PBS. In the second stage (i.e., after the first 2 days), all three types of hydrogels exhibited a slow but stable DOX release until the end of the in vitro release tests. The slow-release behavior in this phase may be attributed to the slow diffusion of DOX molecules from the interior of hydrogel samples to the immersing liquid and the biodegradation of the hydrogel samples themselves. DOX was present in the hydrochloride form, and its favorable hydrophilic property facilitated its homogeneous integration with the hydrogel, leading to enhanced diffusion throughout the release mechanism [56]. For the hydrogel blend with 100 μg/mL DOX, under the physiological condition (PBS, pH 7.4, 37 °C), the hydrogel blend released up to 59.61% of total DOX within 24 h. At 21 days, its cumulative release of DOX was 69.09%. As for the hydrogel blend with 200 μg/mL DOX, DOX release was 53.66% at 24 h and 60.23% at 21 days, whereas the hydrogel blend with 500 μg/mL DOX showed 54.45% release at 21 days. As can be seen, the CSMA-8MC hydrogel with a higher DOX loading showed a smaller initial rapid release and a slower percentage release at the second stage of release (Figure 9a). Shi et al. also noted similar DOX release kinetics in the synthesized poly(ethylene glycol)-block-poly(γ-ethyl-l-glutamate) hydrogels [57]. In their study, the burst release of DOX within the initial 48 h was attributed to the swift diffusion of the drug. The subsequent sustained release over the following 288 h was believed to be a consequence of both DOX diffusion and hydrogel erosion. They observed that the cumulative release reached approximately 57% at the end of 336 h. Wang et al. also found that the release behavior of DOX from DOX@o-phthalaldehyde-terminated 4-armed poly(ethylene glycol) hydrogel exhibited an initial burst phase, followed by a gradual and continuous release [58]. The release dynamics of DOX were predominantly governed by the diffusion of drug molecules. In another study by Xu et al., DOX-loaded gelatin/gellan gum exhibited a release of nearly 50% of DOX within 8 h [59]. This phenomenon was explained by the significant release of DOX from the outer layer of the hydrogel matrix. Therefore, our observations in the current study generally agreed with the findings reported by other researchers. However, in terms of the absolute amount of the drug released, the hydrogel blend with the highest DOX content provided a much larger amount of DOX at both the initial rapid release phase and the second phase of stable and sustained release, as shown in Figure 9b (143.37 μg at 21 day for hydrogel with 500 μg/mL DOX, compared to 65.48 mg at 21 day for hydrogel with 200 μg/mL DOX scaffolds and 35.43 μg at 21 day for hydrogel with 100 μg/mL DOX). These results are consistent with Fick’s diffusion law, where the driving force for diffusion of a species is generated by its concentration gradient [6]. As the DOX loading increased in the hydrogel blend, the DOX concentration difference between the hydrogel blend and immersion liquid increased, and hence, the mass of DOX diffused or released from the hydrogel blend increased. Compared to hydrogels that released all DOX contents within a short period of time (e.g., within 4 days [60]), the hydrogels prepared in the current study had the ability to deliver DOX over a long period of time, which increased the probability of eliminating persistent cancer cells. Additionally, compared to the limited capability of drug loading via physical adsorption or chemical conjugation, the direct blending of drugs during hydrogel ink preparation is more efficient and enables much higher drug loadings, thereby providing enhanced therapeutic effects and minimizing potential drug waste. DOX-containing hydrogels exhibited a red color, which reduced the efficiency of UV crosslinking, and hence, caused a decrease in the mechanical properties of the hydrogel (Figure S6). Lai et al. also reported that the black graphene oxide nanoparticles in their prepared hydrogel scaffolds provided a UV-shading effect [61]. Further studies will be conducted to clarify the detailed mechanism. Considering the diffusion efficiency of DOX and mechanical properties of the hydrogel blend scaffolds, the 200 μg/mL DOX concentration was chosen for constructing new scaffolds in subsequent bioprinting experiments.

**Figure 10:**
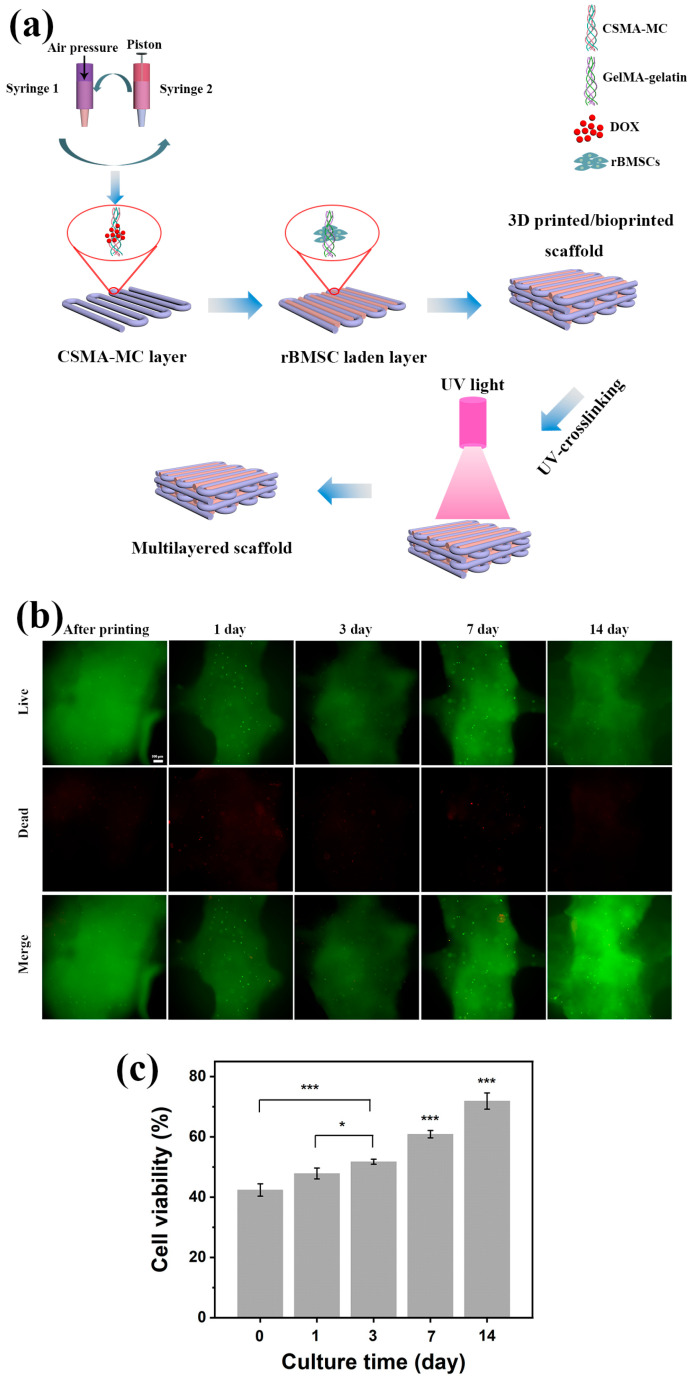
(**a**) Illustration for the fabrication of drug-loaded and cell-laden multilayered hydrogel scaffolds; (**b**) fluorescence images of bioprinted cell-laden scaffolds at different culture times (scale bar: 200 μm; green: live cells, red: dead cells); and (**c**) cell viability in multilayered scaffolds at different culture times (* *p* < 0.05, ** *p* < 0.01, and *** *p* < 0.001).

Three-dimensional bioprinting enables the creation of a cellular environment that closely mimics the conditions found in the human body [62,63]. This 3D cellular environment allows for the observation of distinct drug responses that differ from those observed in traditional 2D cell cultures. Mesenchymal stem cells (MSCs) are an ideal cell source for tissue regeneration owing to their ubiquitous distribution across various tissues and their remarkable capacity to differentiate into diverse terminal cell lineages [64]. In skin regeneration, MSCs have the ability to augment cell proliferation and angiogenesis, while simultaneously diminishing the inflammatory reaction in areas of skin that have been damaged [65]. To avoid any detrimental effects of loading DOX in hydrogel scaffolds on normal cells, experiments were conducted by using rBMSCs in bioprinting so as to evaluate the effects of DOX. GelMA/gelatin blends are temperature-sensitive, having thermally reversible polymer chains in a randomly curled conformation above 26–30 °C, which structurally transforms into a triple helix below 25 °C due to hydrogen bonding [26]. This means that living cells can be incorporated into the ink in the liquid state and the cell-containing bioink transforms into a hydrogel at or above 25 °C to facilitate printing with the printed cell-laden structure staying stable for subsequent photo-crosslinking. Therefore, GelMA/gelatin has been commonly used in bioprinting. CSMA-8MC@200DOX with satisfactory printability, mechanical properties, and drug-release behavior was selected for 3D printing to form integrated structures for melanoma therapy. As shown in Figure 10a, a layer of CSMA-8MC@200DOX scaffold was firstly 3D-printed, followed by the bioprinting of a layer of rBMSC-laden GelMA/gelatin scaffold, with the subsequent layer being 90° to the previous layer. After the construction of the multilayered scaffold, the live/dead assay was used to investigate the viability of rBMSCs in the scaffold, and the results are shown in Figure 10b (green: live cells, red: dead cells). The initial viability of rBMSCs in the scaffolds was found to be 42.38% immediately after printing (Figure 10c). This reduced viability could be attributed to the exposure of cells to shear stress while passing through the nozzle of the printing head during the printing process. Furthermore, UV crosslinking for a duration of 2 min subsequent to printing may have also contributed to the decline in cell viability. In a study conducted by Zhang et al., gelatin and alginate were employed as bioinks for bioprinting [66]. Similar to our printing protocol, the cell-containing bioink required pre-cooling by refrigeration for approximately 5 min to achieve a gel state prior to bioprinting. The cell survival in their study was found to be 54.72%. They hypothesized that the diminished cell viability could be attributed to the combined effects of shear force, low temperature, and extended printing duration. In addition to these factors, while DOX has a demonstrated efficacy in treating various solid tumors and hematological malignancies, its therapeutic action is not selective and, hence, affects both tumor cells and normal cells. The combination of all these factors had led to a low initial cell viability after printing in the current study. However, despite the low cell viability during the initial days of culture, there was a good increase subsequently in cell proliferation when the culture time increased (Figure 10c). GG has been extensively investigated as a type of bioink. In a study by Lai et al., the MTT test was employed to assess the proliferation of rBMSCs within bioprinted GG hydrogels. The results revealed a positive correlation between MTT absorbance levels and the duration of cell culture, suggesting the supportive role of GG ink in promoting the proliferation of rBMSCs. After 14 days of culture, the cell viability in our prepared scaffolds was restored to 71.87%. This observation suggested that the multilayer hydrogel scaffolds could provide a conducive microenvironment for treating melanoma resection-induced skin defects. Peng et al. prepared sodium alginate/gelatin/human corneal fibroblast cell line bioink [67]. The cell viability after printing was about 95%, and the cell viability decreased to about 70% after 7 days of culture. However, using a bioink of similar polymer matrix, the sodium alginate/gelatin/Pluronic F127 hydrogel encapsulated with an osteoblast-like cell line (MG-63) was bioprinted [68]. The cell viability was about 50% after bioprinting, and the cell viability increased by about 5% after 7 days of culture. Although the ink systems in these two separate investigations were similar, the cell viability results were very different. These results indicated that, post-bioprinting, the cell viability could be influenced by a range of factors, including the composition of the ink material, volume percentages of various material components in the ink, and the specific cell type employed. In our initial in vitro biological experiments, it was found that the survival rate of rBMSCs in bioprinted structures could be improved over the cell culture period (14 days). However, further experiments are needed to evaluate the long-term viability and proliferation of encapsulated cells.

A previous work by Wang et al. confirmed that low doses of DOX had little effect on the cell viability of rBMSCs [11]. A total of 200 μg/mL DOX is a medium-level concentration reported in the open literature (compared to 2.5 mg/mL or 3 mg/mL) for drug release studies [6,69,70], and it did not appear that this DOX concentration affected the long-term viability of rBMSCs in the current study. This result suggests that our hydrogel scaffold-based drug release system could be used to provide integrative melanoma therapy.

It is important to bear in mind that DOX, a widely used anticancer drug in chemotherapy, was selected as a model drug for investigations in current study. However, melanoma is known to develop resistance to drugs, resulting in the use of multiple drugs, including metformin [71], indocyanine green [72], etc., for achieving combinational chemotherapy. Further studies are needed to explore the concurrent release of multiple drugs from 3D-printed drug-loaded hydrogel scaffolds.

## 4. Conclusions

Three-dimensionally printed CSMA-MC hydrogel blend tissue engineering scaffolds possessed remarkable properties and may be employed to provide integrative tumor therapy for melanoma. The addition of MC to the CSMA hydrogel resulted in the good printability of inks, which allowed for the printing of multilayered lattice structures that did not collapse and ensured the structural integrity of the printed structures. The entangled polymer networks in the hydrogel blends could withstand stresses from external loads due to the interactions between CSMA and MC. The compression modulus of the hydrogels prepared exhibited a range from 3.4 to 9.1 kPa. CSMA-8MC was selected as the DDS owing to its elevated compression modulus and rapid crosslinking time. A concentration of 200 μg/mL was selected to avoid the UV shading of red DOX. DOX could be incorporated and subsequently released from the hydrogels in a sustained manner. By the 21st day, approximately 60.23% of the DOX had been successfully released. In addition, DOX-loaded CSMA-MC scaffolds as a controlled DDS could support the proliferation of rBMSCs, thereby achieving both integrative tumor therapy for melanoma and the tissue regeneration of skin.

## Figures and Tables

**Table 1:** CSMA/MC hydrogel blends for 3D printing.

Hydrogel Blend Composition (% *w*/*v*)	Designation
1% CSMA, 6% MC	CSMA-6MC
1% CSMA, 8% MC	CSMA-8MC
1% CSMA, 10% MC	CSMA-10MC
1% CSMA, 12% MC	CSMA-12MC

## Data Availability

The original contributions presented in the study are included in the article/Supplementary Material, further inquiries can be directed to the corresponding author.

## Supplementary Materials

The following supporting information can be downloaded at: https://www.mdpi.com/article/10.3390/jfb15120381/s1, Figure S1. (a) Chemical structures of gelatin and GelMA, and (b) ^1^H NMR spectra of gelatin and GelMA; Figure S2. (a) XRD patterns and (b) TGA curves of CSMA and MC raw materials; Figure S3. Photos of 3D-printed 10-layer hydrogel blend grid structures from the front view; Figure S4. (a) Compressive stress–strain curves of hydrogel samples with different MC concentrations. (Solid curves were typical curves of all tested samples). (b) Compressive stress at yield for CSMA-6MC and CSMA-8MC; Figure S5. n_e_ of blend hydrogels obtained from a frequency sweep; Figure S6. Compressive strain–stress curve of hydrogel blends with different DOX concentrations. Reference [73] are cited in the supplementary materials.
