# Supplementary material for: Three-Dimensional Printing of Hydrogel Blend Tissue Engineering Scaffolds with In Situ Delivery of Anticancer Drug for Treating Melanoma Resection-Induced Tissue Defects
Source: J Funct Biomater. 2024 Dec 18;15(12):381. doi: 10.3390/jfb15120381 (PMC11678273; doi:10.3390/jfb15120381)
Supplement: Supplementary file 1 [file jfb-15-00381-s001.zip › jfb-3283461-supplementary.pdf]

Journal of *Functional Biomaterials*

MDPI

Supplementary Information

Three-Dimensional Printing of Hydrogel Blend Tissue Engineering Scaffolds with in Situ Delivery of Anticancer Drug for Treating Melanoma Resection-Induced Tissue Defects

Xiao-Die Chen [1], Xin-Yang Zhang [1], Han-Qi Zhu [1], Helen H. Lu [2] and Min Wang [1,*]

[1] Department of Mechanical Engineering, The University of Hong Kong, Pokfulam Road, Hong Kong, China; xdchen@connect.hku.hk (X.C.); u3008377@connect.hku.hk (X.Z.); zhuhanqi@hku.hk (H.Z.)
[2] Department of Biomedical Engineering, Columbia University, 1210 Amsterdam Avenue, New York, NY 10027, USA; hl2052@columbia.edu
* Correspondence: memwang@hku.hk; Tel: +852-3917-7903; Fax: +852-2858-5415

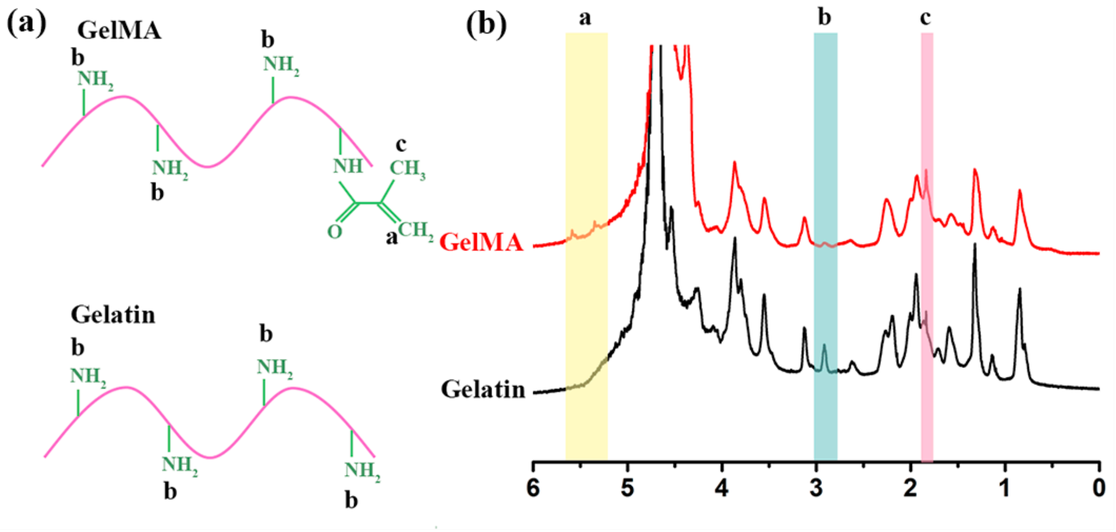

Figure S1. (a) Chemical structures of gelatin and GelMA, and (b)^{1}H NMR spectra of gelatin and GelMA.

GelMA was synthesized according to Lai's work with minor modifications [1]. Briefly, 10 g gelatin was dissolved in 100 ml PBS at 50 °C. 6 ml MA was then added into the gelatin solution and stirred for 3 to 4 h. Afterwards, the mixture was dialyzed (MW 12 KDa cut-off) for a week at 50 °C. Finally, the solution was freeze-dried and kept at -20 °C until use.

^{1}H NMR spectra were recorded on a Bruker Avance III 400 spectrometer (400 MHz) at 25 °C, using D2O(δ(^{1}H) = 4.79 ppm as solvent. The degree of substitution (DS) of GelMA was calculated to be 60.69% (Figure S1).

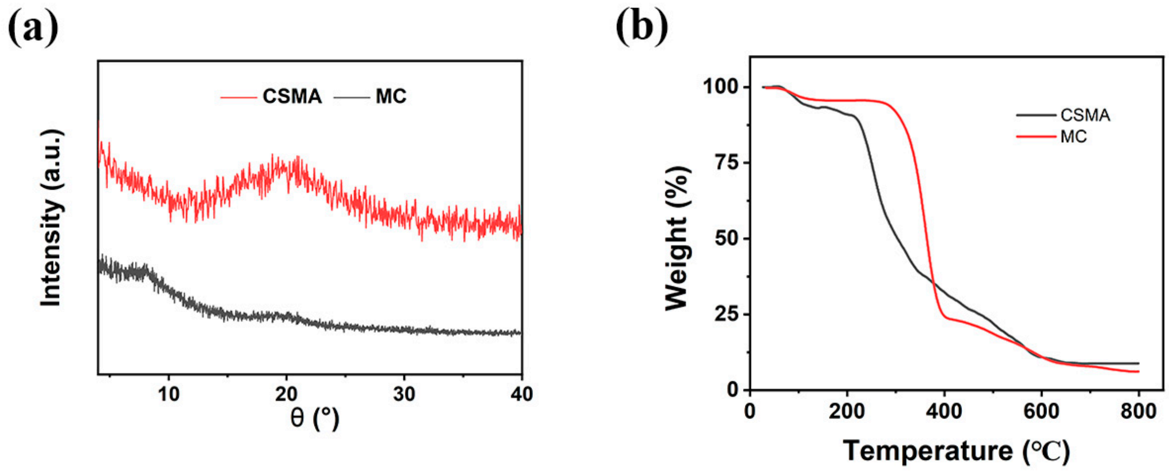

Figure S2. a) XRD patterns and (b) TGA curves of CSMA and MC raw materials.

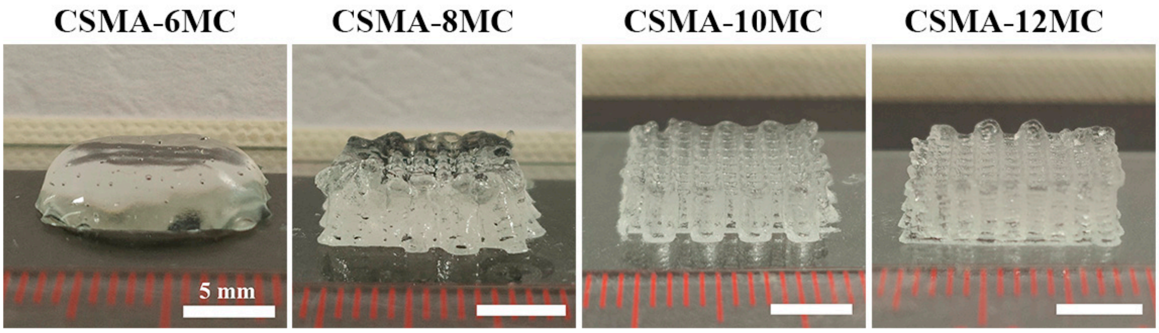

Figure S3. Front views of 3D printed 10-layer hydrogel blend grid structures using different inks.

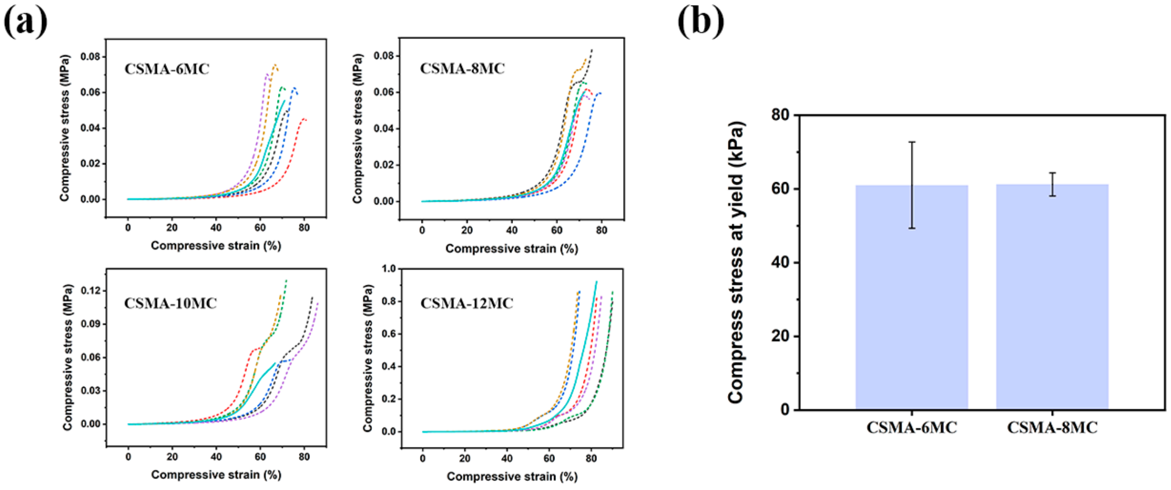

Figure S4. a) Compressive stress-strain curves of hydrogel samples with different MC concentrations. (The dash curves were individual samples, and the solid curves were the average curves of all tested samples) (b) Compressive stress at yield for CSMA-6MC and CSMA-8MC.

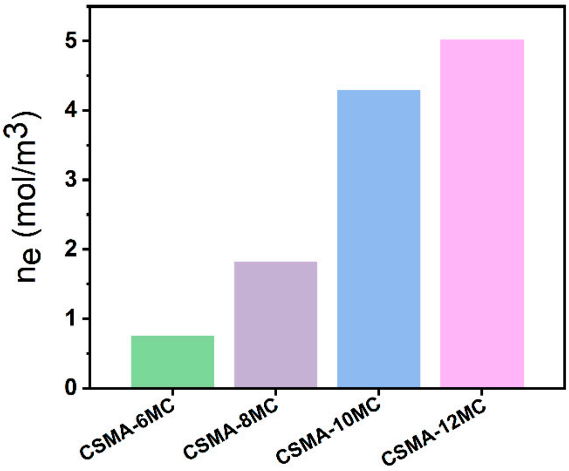

Figure S5. n_e values of blend hydrogels calculated from frequency sweep results of rheological tests.

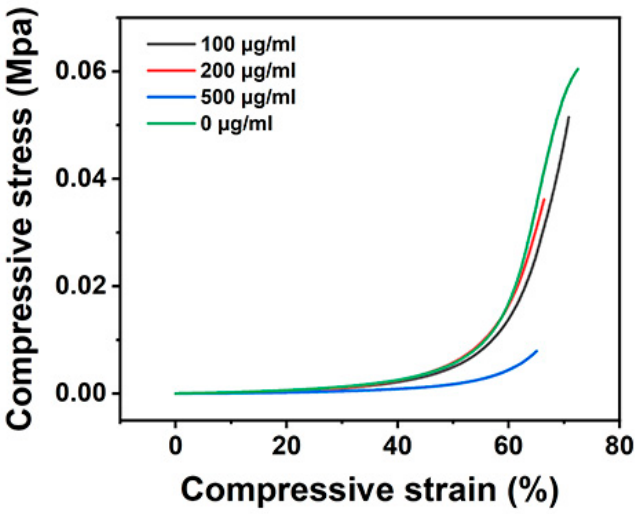

Figure S6. Compressive strain-stress curve of hydrogel blends having different DOX concentrations.

Reference

1. Lai J., J. Li, and M. Wang, *3D printed porous tissue engineering scaffolds with the self-folding ability and controlled release of growth factor.* MRS Communications, 2020. **10**(4): p. 579-586.
